# Individualized Prediction of Drug Resistance in People with Post-Stroke Epilepsy: A Retrospective Study

**DOI:** 10.3390/jcm12113610

**Published:** 2023-05-23

**Authors:** Simona Lattanzi, Stefano Meletti, Eugen Trinka, Francesco Brigo, Gianni Turcato, Claudia Rinaldi, Claudia Cagnetti, Nicoletta Foschi, Serena Broggi, Davide Norata, Mauro Silvestrini

**Affiliations:** 1 Neurological Clinic, Department of Experimental and Clinical Medicine, Marche Polytechnic University, 60121 Ancona, Italy; 2 Neurology Unit, OCB Hospital, AOU Modena, 41125 Modena, Italy; 3 Department of Biomedical, Metabolic and Neural Science, Center for Neuroscience and Neurotechnology, University of Modena and Reggio Emilia, 41121 Modena, Italy; 4 Department of Neurology, Christian Doppler Klinik, Paracelsus Medical University, 5020 Salzburg, Austria; 5 Center for Cognitive Neuroscience, 5020 Salzburg, Austria; 6 Public Health, Health Services Research and HTA, University for Health Sciences, Medical Informatics and Technology, 6060 Hall in Tirol, Austria; 7 Emergency Department, “Franz Tappeiner” Hospital, 39012 Merano, Italy; 8 Department of Internal Medicine, Hospital of Santorso, 36014 Santorso, Italy

**Keywords:** brain infarct, cerebral hemorrhage, nomogram, seizures, stroke

## Abstract

Background: The study aimed to develop a model and build a nomogram to predict the probability of drug resistance in people with post-stroke epilepsy (PSE). Methods: Subjects with epilepsy secondary to ischemic stroke or spontaneous intracerebral hemorrhage were included. The study outcome was the occurrence of drug-resistant epilepsy defined according to International League Against Epilepsy criteria. Results: One hundred and sixty-four subjects with PSE were included and 32 (19.5%) were found to be drug-resistant. Five variables were identified as independent predictors of drug resistance and were included in the nomogram: age at stroke onset (odds ratio (OR): 0.941, 95% confidence interval (CI) 0.907–0.977), intracerebral hemorrhage (OR: 6.292, 95% CI 1.957–20.233), severe stroke (OR: 4.727, 95% CI 1.573–14.203), latency of PSE (>12 months, reference; 7–12 months, OR: 4.509, 95% CI 1.335–15.228; 0–6 months, OR: 99.099, 95% CI 14.873–660.272), and status epilepticus at epilepsy onset (OR: 14.127, 95% CI 2.540–78.564). The area under the receiver operating characteristic curve of the nomogram was 0.893 (95% CI: 0.832–0.956). Conclusions: Great variability exists in the risk of drug resistance in people with PSE. A nomogram based on a set of readily available clinical variables may represent a practical tool for an individualized prediction of drug-resistant PSE.

## 1. Introduction

Stroke is a common condition with a worldwide estimated incidence of more than 15 million cases every year [1]. Post-stroke epilepsy (PSE), defined as the occurrence of one or more unprovoked epileptic seizures at least seven days after stroke onset, is an impactful consequence of stroke. It develops in at least 4–6% of patients with stroke and accounts for 50% of all cases of newly diagnosed epilepsy among people aged 60 years and older [2]. Although PSE generally has a good response to antiseizure medicines (ASMs), around 20% of the subjects are drug-resistant [3].

Prognostic prediction models estimate the individual risk of future outcome, which is conditional on the values of multiple prognostic factors. Scoring systems are available to estimate the risk of epilepsy after stroke, such as the SeLECT and CAVE scores. Contrariwise, only a few studies have used multivariable models to estimate the likelihood of treatment response [3,4,5,6], and there is still the need to create tools to easily predict drug-resistant PSE.

Nomograms are statistical tools that allow one to take into account numerous risk factors and perform a comprehensive risk assessment for a specific endpoint in a particular subject based on unique demographic and disease variables. These models are useful for clinical counselling and decision making and are increasingly being applied to epilepsy.

The aim of this study was to develop a model and build a nomogram to predict the probability of drug resistance in people with PSE.

## 2. Material and Methods

### 2.1. Participants, Study Outcome and Predictors

We retrospectively identified adults referred to the Epilepsy Center of the Marche Polytechnic University who were diagnosed with PSE, i.e., epilepsy secondary to ischemic stroke or spontaneous intracerebral hemorrhage, and no history of seizures before the stroke [6]. The epilepsy center is a public setting center with three full-time dedicated epileptologists. Seizures that occurred within 7 days of stroke onset were considered as acute symptomatic seizures and those that occurred after 7 days were considered as unprovoked seizures; PSE was diagnosed as the occurrence of one or more unprovoked seizure [7].

The study outcome was drug-resistant epilepsy at any time during follow-up. Subjects were considered drug-resistant if they continued to experience seizures despite two adequate trials of tolerated and appropriately chosen and used ASM schedules, according to the current definition of drug-resistant epilepsy [8]. Participants who did not receive appropriate ASM schedules for at least 1 year or had a follow-up after <12 months were excluded to allow a consistent definition of drug-resistant epilepsy according to the consensus that the seizure-free duration should be at least 12 months [8].

The candidate predictors considered for model development were sex, age at stroke onset, stroke type (ischemic stroke, intracerebral hemorrhage), stroke severity (severe stroke defined as a National Institutes of Health Stroke Scale score at stroke onset ≥ 16 according to medical convention) [9], the occurrence of acute symptomatic post-stroke seizures, the latency of PSE, defined as the time frame between stroke onset and the occurrence of the first unprovoked post-stroke seizure (0–6, 7–12, >12 months), status epilepticus (SE) at PSE onset, and seizure types (focal onset, focal-to-bilateral tonic-clonic, generalized or unknown onset). These variables were selected for their known association with pharmaco-resistance in people with PSE and the ease with which they can be obtained during clinical assessment in most epilepsy centers [3,4,5,6]. All information about clinical predictors and seizure occurrence was obtained from medical records.

### 2.2. Statistical Analysis

Values were presented as mean (SD) or median (interquartile range) for normally distributed and not normally distributed continuous variables and as the number (percent) of patients for categorical variables. A logistic regression model was fitted using a backward stepwise method that included the pre-established variables (i.e., sex, age at stroke onset, stroke type, severe stroke, acute symptomatic post-stroke seizures, latency of PSE, SE at epilepsy onset, and seizure type) to identify the independent predictors of drug resistance. Regression coefficients with standard error and odds ratios (OR) with 95% confidence intervals (CIs) were calculated. The weight of any variable in the prediction model was obtained via the regression coefficients, which were used to generate the nomogram. The discriminatory ability of the nomogram was evaluated using the area under the receiver operating characteristic curve (AUROC). As multivariate predictor models tend to be overfitted to the original sample, the model was internally validated through a bootstrap of 5000 samples and an error-corrected AUROC was estimated. The calibration was evaluated by plotting predicted against observed outcomes and by using the Hosmer–Lemeshow test; in well-calibrated models, the predictions should fall on a 45-degree diagonal line. Decision curve analysis (DCA) was performed to quantify the net benefits at different threshold probabilities and determine the clinical utility of the model; a good model usually yields a high net benefit over a wide range of threshold probabilities in the DCA curve [10]. Results were considered significant for *p* values < 0.05 (two-sided). Data analysis was performed using the STATA/IC 13.1 statistical package. The present study was reported following the Transparent Reporting of a multivariable prediction model for Individual Prognosis or Diagnosis (TRIPOD) guidelines [11].

## 3. Results

Of 178 patients with PSE initially identified at the center, 14 patients were excluded due to follow-up being <12 months. Accordingly, 164 patients with PSE were included in the analysis and 32 (19.5%) were found to be drug-resistant. The median duration of the follow-up was 5.5 (3.0–11.5) years for drug-resistant and 5.0 (3.0–8.5) years for non-drug-resistant participants (*p* = 0.612; Mann–Whitney test).

The mean age of the participants at stroke onset was 56.9 (14.9) years, and 107 (65.2%) were male. Cerebral infarct occurred in 105 (64.0%) and intracerebral hemorrhage in 59 (36.0%) subjects; severe strokes accounted for 80/164 (48.8%) cases. The median latency from stroke to PSE was 18 (11–38) months, and SE was the first manifestation of PSE in 12 (7.3%) cases. Baseline characteristics of the study participants are reported in Table 1.

Descriptive statistics and unadjusted associations between each predictor and drug-resistant PSE are summarized in Table 2. Younger age at stroke onset, spontaneous cerebral hemorrhage, severe stroke, shorter latency of PSE, and SE at epilepsy onset were associated with drug resistance in the unadjusted model.

Of the eight pre-established variables entered into the logistic regression model, five were independent predictors: age at stroke onset (OR: 0.941, 95% CI 0.907–0.977), stroke type (ischemic stroke, reference; intracerebral hemorrhage OR: 6.292, 95% CI 1.957–20.233), stroke severity at stroke onset (no severe stroke, reference; severe stroke OR: 4.727, 95% CI 1.573–14.203), latency of PSE (>12 months, reference; 7–12 months, OR 4.509, 95% CI 1.335–15.228; 0–6 months, OR 99.099, 95% CI 14.873–660.272), and SE at epilepsy onset (no, reference; yes, OR 14.127, 95% CI 2.540–78.564) (Table 3).

**Figure 1:**
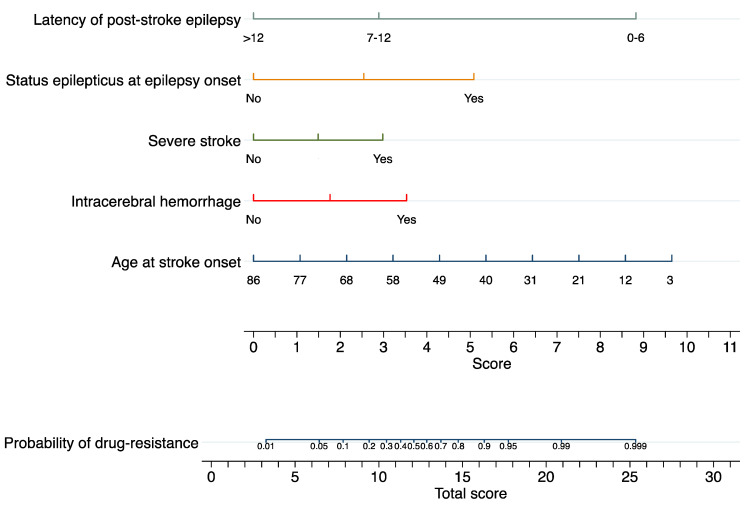
Prognostic nomogram to predict drug-resistant post-stroke epilepsy. To use the nomogram, locate the subject’s position on the scale associated with each predictor. Each predictor receives points on the preliminary score by drawing a vertical line between the predictor line and score line. The total score is the sum of the points assigned to each predictor. The probability of drug-resistant post-stroke epilepsy is obtained by drawing a vertical line between the total score (bottom) axis and the probability line. The latency of post-stroke epilepsy was defined as the time interval (months) between stroke onset and the occurrence of the first unprovoked post-stroke seizure (0–6, 7–12, >12 months); severe stroke was defined as a National Institutes of Health Stroke Scale score at stroke onset ≥ 16.

**Figure 2:**
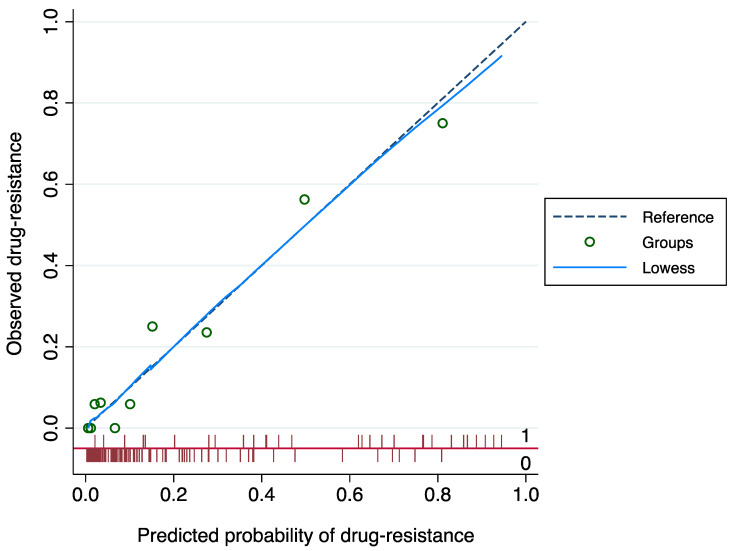
Model calibration. Calibration plot of model predicting drug-resistant post-stroke epilepsy. The model was applied to the cohort and predictions generated from the model were plotted against actual outcomes.

**Figure 3:**
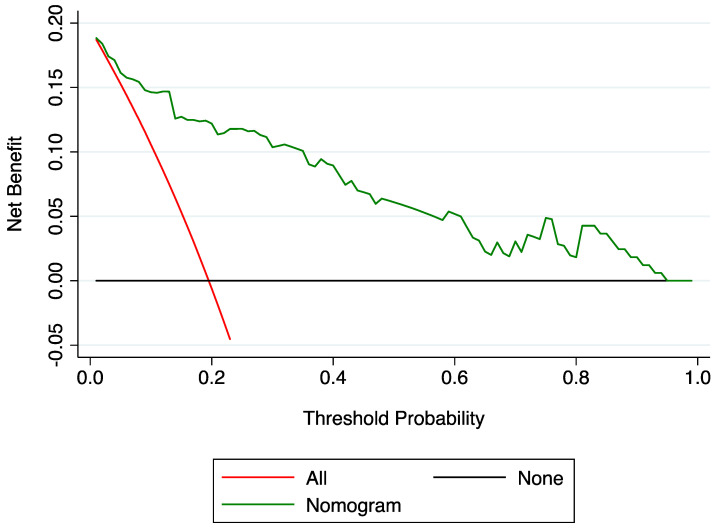
Decision curve analysis for the nomogram model to predict drug-resistant post-stroke epilepsy. A decision curve analysis for the nomogram model to predict drug-resistant post-stroke epilepsy. The x-axis indicates the threshold probability, and the y-axis indicates the net benefit. The black line represents the net benefit assuming that no subjects have drug-resistant post-stroke epilepsy. The red line shows the net benefit assuming that all subjects have drug-resistant post-stroke epilepsy. As shown by the green curve, when the threshold probability is <0.95, using the nomogram prediction model yields significant net clinical benefits for subjects.

The nomogram to predict drug-resistant PSE is shown in Figure 1. The AUROC of the nomogram was 0.894 (95% CI: 0.833–0.956); the bootstrap-corrected C-statistic after internal validation was 0.893 (95% CI: 0.832–0.956). The calibration plot is displayed in Figure 2 and showed a good consistency between the predicted probability and observed frequency of drug resistance; the Hosmer–Lemeshow goodness-of-fit test comparing the predicted and observed rates of drug-resistant PSE revealed good calibration of the model (*p* = 0.717). The DCA to evaluate the clinical usefulness of the model is illustrated in Figure 3; subjects with PSE gain clinical benefit from using the nomogram prediction model when the risk threshold probability is <95%.

## 4. Discussion

Drug resistance in epilepsy is a multifactorial phenomenon and both patient-related and disease-related variables play a role. This study proposed a multivariable model that concurrently accounts for multiple predictors and estimates the probability of drug-resistance for an individual patient with PSE based on their distinctive clinical characteristics. The nomogram developed represents a practical tool for a quick individualized prediction of drug-resistant PSE using a set of readily available clinical information.

Age at stroke onset and time from stroke to PSE were the variables associated with the greatest relative importance among the predictors, as can be judged by the length of the lines within the nomogram. Consistent with the existing literature, younger age at stroke onset was linked with a higher probability of drug-resistant PSE, and older age acted as a protective factor. In a retrospective analysis of adults with epilepsy following a non-traumatic intracerebral hemorrhage, responders were significantly older than resistant participants [4]. Burneo et al. performed a population-based retrospective cohort study using administrative data to explore factors associated with refractory epilepsy among stroke survivors [5]. Out of 210 participants with PSE, those who became pharmacologically refractory were more likely to be younger [5]. Smooth neuronal plasticity with increasing age may result in reduced epileptogenicity and explain the lower risk of drug-resistant epilepsy.

Our prior research indicated that the time from stroke to PSE is inversely associated with the likelihood of pharmaco-resistance, with a shorter latency being related to a higher risk of uncontrolled epilepsy: the risk of drug-resistance was highest when epilepsy developed within the first few months after stroke and decreased progressively with a steeper decline when epilepsy onset occurred after the first year [6]. This nonlinear dose–response relationship can be observed in the nomogram via the greater weight that a latency of 6 months or shorter has compared to a latency of 7 months or longer. The evidence that the latent period may inform the course of epilepsy supports the hypothesis of epileptogenesis as a progressive rather than stepwise process, which starts after the brain insult and continues into chronic epilepsy through dynamic circuitry reorganization [12].

The relationship between SE as the first clinical manifestation of PSE and the risk of drug resistance can be interpreted bidirectionally. On one hand, SE can trigger modifications in the organization of neuronal networks by means of inflammatory reactions, disruption of the blood–brain barrier, and reactive synaptogenesis, which ultimately contribute to drug refractoriness [13]. On the other hand, SE may already be the clinical hallmark of changes in the neuronal networks that characterize refractory epilepsy within the frame of the “intrinsic severity” hypothesis of drug resistance. Of note, the occurrence of SE has been shown to be associated with seizure intractability in other forms of epilepsies [14].

Alterations in the properties of neurotransmitter receptors and ion channels, overexpression of efflux transporters and sprouting of synaptic connections occur at brain level in response to acute vascular injuries. While these adaptations are key determinants of the recovery from cerebral damage, they can also result in aberrant plasticity, contribute to the hyper-synchronization of neuronal circuits, and limit the access of ASMs to neuronal targets [15]. The entity of the injury and the presence of blood derivatives in the brain can act as crucial modifiers of these reactive changes and enhance those maladaptive responses that ultimately contribute to drug resistance [14,16]. These events may contribute to explaining the higher probability of pharmaco-resistance in people with severe stroke and intracerebral hemorrhage.

This study has the merit of having developed a nomogram that is easy to use and has very good performance in predicting drug resistance in people with PSE. The model was internally validated and had excellent discrimination, being able to correctly classify participants as drug-resistant and non-drug-resistant 89% of the time. The calibration plot indicated that predictions based on the model were reliable representations of actual risks. The inclusion of variables that are easily ascertainable during standard clinical practice makes it suitable for routine clinical use. In parallel, a few shortcomings need to be considered. The main limitation is the lack of the external validation of the model in an independent cohort. Subjects recruited at an epilepsy clinic may have represented a preselected cohort and the retrospective analysis of data may have introduced potential sources of biases with regard to the diagnosis of PSE, status epilepticus, and drug-resistant epilepsy and limited the opportunity to evaluate the actual compliance with treatment over time. Considering that drug resistance is not a fixed state, a prospective study would be needed to explore the patterns of relapse and remission during follow-up. The validation of the nomogram in larger cohorts is warranted to confirm the current findings and model performance. Further studies could also evaluate whether the adjunct of additional variables, including but not limited to the frequency and duration of seizures, EEG, and neuroimaging features, may improve the predictive accuracy of the model. Although cortical involvement has not been shown to be a risk factor of drug resistance [3], its possible relationship with the development of drug-resistant PSE needs to be further investigated, as well as the potential role of the location of stroke, including the involvement of the temporal lobe.

Substantial variability may exist in the risk of drug resistance among people with PSE. In this regard, the early identification of factors associated with drug-resistant epilepsy represents one main area of research [17]. The ability to identify people at high risk of poor seizure control, ideally at the time of treatment initiation, can hold great clinical utility. It can counsel people with epilepsy and their caregivers about the expected disease course, guide clinicians in the decision regarding the most appropriate treatment pathway and inform the healthcare system about the resources needed when stroke is accompanied by epilepsy development. People at high risk of drug-resistant PSE can be monitored more closely and regularly, and they can be referred to specialized epilepsy centers for further evaluation and treatment [18]. The use of predictive models may, hence, ensure that people with a high risk of drug resistance receive the best care, including more personalized and aggressive interventions, potentially limiting the morbidity and mortality associated with drug-resistant epilepsy. A reliable nomogram based on a few readily available variables may represent a useful tool to provide personalized predictions in an easy-to-use manner, improve counselling, and increase clinician confidence in risk assessment.

## Figures and Tables

**Table 1:** Baseline characteristics of study participants.

	Participants (*n* = 164)
Male sex	107 (65.2)
Age at stroke onset	56.9 (14.9)
Familiar history of seizures	6 (3.7)
Hypertension	112 (68.3)
Diabetes mellitus	31 (18.9)
Dyslipidemia	63 (38.4)
Atrial fibrillation	23 (14.0)
Coronary heart disease	30 (18.3)
Stroke type	
Cerebral infarct	105 (64.0)
Intra-cerebral hemorrhage	59 (36.0)
^a^ Stroke severity	
Mild to moderate	84 (51.2)
Severe	80 (48.8)
^b^ Acute symptomatic post-stroke seizures	25 (15.2)
Status epilepticus at epilepsy onset	12 (7.3)
Seizure type	
Focal onset	83 (50.6)
Focal-to-bilateral tonic clonic	58 (35.4)
Generalized or unknown onset	23 (14.0)
^c^ Epilepsy latency, months	18 (11–38)

Data are mean (SD) or median [IQR] for continuous variables and *n* (%) for categorical variables. ^a^ Stroke was defined as mild to moderate if the NIHSS score at stroke onset was <16 and severe if initial the NIHSS score was ≥16. ^b^ Seizures occurring within 7 days of stroke onset. ^c^ The latency of epilepsy was defined as the time interval between stroke onset and the occurrence of the first unprovoked post-stroke seizure. Abbreviations: IQR = interquartile range, NIHSS = National Institutes of Health Stroke Scale, SD = standard deviation.

**Table 2:** Descriptive statistics and unadjusted association between each predictor and drug-resistant post-stroke epilepsy.

	Drug Responsive (*n* = 132)	Drug-Resistant (*n* = 32)	Unadjusted OR (95% CI)	*p* Value
Male, *n* (%)	87 (65.9)	20 (62.5)	0.86 (0.39–1.92)	0.717
Age at stroke onset, y, mean (SD)	58.1 (14.8)	52.0 (14.5)	0.97 (0.95–0.99)	0.041
Intracerebral hemorrhage, *n* (%)	41 (31.1)	18 (56.3)	2.85 (1.30–6.29)	0.009
Severe stroke, *n* (%)	55 (41.7)	25 (78.1)	5.00 (2.02–12.38)	0.001
Acute symptomatic post-stroke seizures, *n* (%)	18 (13.6)	7 (21.9)	1.77 (0.67–4.70)	0.249
Latency of post-stroke epilepsy				
0–6 months, *n* (%)	3 (2.3)	7 (21.9)	16.02 (3.73–68.78)	<0.001
7–12 months, *n* (%)	26 (19.7)	10 (31.2)	2.64 (1.06–6.55)	0.036
>12 months, *n* (%)	103 (78.0)	15 (46.9)	1.00 (reference)	-
Status epilepticus at epilepsy onset, *n* (%)	5 (3.8)	7 (21.9)	7.11 (2.09–24.21)	0.002
Seizure type				
Focal onset only	69 (52.3)	14 (43.8)	1.00 (reference)	-
Focal-to-bilateral tonic-clonic	41 (31.1)	17 (53.1)	2.04 (0.91–4.58)	0.082
Generalized or unknown onset	22 (16.7)	1 (3.1)	0.22 (0.03–1.80)	0.160

Severe stroke was defined as a National Institutes of Health Stroke Scale score at stroke onset ≥ 16. The latency of post-stroke epilepsy was defined as the time interval (months) between stroke onset and the occurrence of the first unprovoked post-stroke seizure (0–6, 7–12, >12 months).

**Table 3:** Predictors of drug-resistant post-stroke epilepsy in the nomogram model according to multivariate logistic regression.

	Regression Coefficient (Standard Error)	Adjusted OR (95% CI)	*p* Value
Age at stroke onset	−0.061 (0.019)	0.941 (0.907–0.977)	0.001
Intracerebral hemorrhage	1.839 (0.596)	6.292 (1.957–20.233)	0.002
Severe stroke	1.553 (0.561)	4.727 (1.573–14.203)	0.006
Latency of post-stroke epilepsy			
0–6 months	4.596 (0.968)	99.099 (14.873–660.272)	<0.001
7–12 months	1.506 (0.621)	4.509 (1.335–15.228)	0.015
>12 months	0 (reference)	1.000 (reference)	-
Status epilepticus at epilepsy onset	2.648 (0.875)	14.127 (2.540–78.564)	0.002

The logistic regression model is: Log_e_ p(x)/[1 − p(x)] = −1.088 − (0.061 × age at stroke onset) + (1.839 × intracerebral hemorrhage) + (1.553 × severe stroke) + (4.596 × latency of post-stroke epilepsy 0–6 months) + (1.506 × latency of post-stroke epilepsy 7–12 months). No significant statistical collinearity was observed for any of the five variables included in the model. Severe stroke was defined as a National Institutes of Health Stroke Scale score at stroke onset ≥16. The latency of post-stroke epilepsy was defined as the time interval (months) between stroke onset and the occurrence of the first unprovoked post-stroke seizure (0–6, 7–12, >12 months).

## Data Availability

Anonymized data will be shared by request from any qualified investigator.
